# Applying bioethical principles to human biomonitoring

**DOI:** 10.1186/1476-069X-7-S1-S8

**Published:** 2008-06-05

**Authors:** Myron Harrison

**Affiliations:** 1 Senior Medical Advisor, Exxon Mobil Corporation, Irving, Texas, USA

## Abstract

Bioethical principles are widely used as a normative framework in areas of human research and medical care. In recent years there has been increasing formalization of their use in public health decisions. The "traditional bioethical principles" are applied in this discussion to the important issue human biomonitoring for environmental exposures. They are: (1) Autonomy – Also known as the "respect for humans" principle, people understand their own best interests; (2) Beneficence – "do good" for people; (3) Nonmaleficence – "do no harm"; (4) Justice – fair distribution of benefits and costs (including risks to health) across stakeholders.

Some of the points made are: (1) There is not a single generic bioethical analysis applicable to the use of human biomonitoring data, each specific use requires a separate deliberation; (2) Using unidentified, population-based biomonitoring information for risk assessment or population surveillance raises fewer bioethical concerns than personally identified biomonitoring information such as employed in health screening; (3) Companies should proactively apply normative bioethical principles when considering the disposition of products and by-products in the environment and humans; (4) There is a need for more engagement by scholars on the bioethical issues raised by the use of biomarkers of exposure; (5) Though our scientific knowledge of biology will continue to increase, there will always be a role for methods or frameworks to resolve substantive disagreements in the meaning of this data that are matters of belief rather than knowledge.

## Introduction

The National Research Council's report on Human Biomonitoring identified bioethical concerns as one of the significant challenges in the use of biomarkers of exposure for both individual and public health decisions [1]. The same need has been identified by the EU Commission and the European Center for Ecotoxicology and Toxicology of Chemicals [2,3].

Similar concerns, though more emotive, emerged in the 1980's with increased testing for genetic biomarkers [4]. These concerns accelerated with the Human Genome Project. Facilitated by significant funding for scholars to examine anticipated ethical, legal and social issues, many of the important conflicts have been reasonably well addressed [5].

Little scholarly attention has been paid to the bioethical issues associated with biomarkers for environmental exposure. Most of the literature is directed to the use of these technologies in the workplace [6,7]. A 1997 publication included a discussion of ethical issues of biomarkers related to environmental exposures – including those for dose [8]. In the NRC publication mentioned above, ethical issues related to biomonitoring are discussed primarily in terms of confidentiality, privacy and nondiscrimination [1]. A more recent article uses bioethical principles to analyze the decision to communicate personal biomonitoring data to those who have been tested [9].

The purpose of this paper is to contribute to a dialogue on concerns surrounding biomarkers of exposure for industrial chemicals. This will be done by:

• First, making a case for the relevance of normative argumentation to these concerns

• Secondly, applying bioethical principles to some specific decisions that are introduced by the use of biomonitoring technologies

### Can bioethics aid decision-making related to human biomarkers of chemical absorption?

Biomonitoring of humans for markers of chemical exposure has burgeoned because of better sensitivity and decreased cost of the analytical techniques. The most visible application of this information is the CDC's "National Exposure Report" [10] which yields valuable insight into chemicals we absorb from air, food, water and personal products. It is difficult to overstate the impact that this information will have on the way we think about and manage chemicals and health risk [11,12]. The information will enable better medical diagnosis and treatment, more accurate risk assessment, improved product safety, and better public health planning.

But, in the near term, there are considerable challenges in the interpretation of biomonitoring data for individuals and for purposes of public health. An example of these challenges is the current public conflict about the predictive value of exposure biomonitoring in terms of individual health risk. Some groups state or strongly imply that the presence in the body of any level of manmade chemicals is evidence of increased risk of harm [13-15]. A conflicting position states that biomonitoring data for "environmental chemicals" is not meaningful in the absence of a proven link between a chemical and a disease. This line of reasoning often concludes that there is currently no justification to perform these analyses.

### What is the crux of wildly differing interpretations of biomonitoring data?

The two extreme interpretations of the value of human biomonitoring described above are not driven by different scientific analyses of the data. Unfortunately, the actual biological knowledge that could support or dispute a link between low concentrations of chemicals and health effects is not available [16]. Those who make statements regarding risk in this area are typically expressing personal beliefs and values – though, often couched in quasi-scientific jargon. Beliefs and values, however sincerely held, are outside the realm of scientific inquiry [17]. Among these important areas of dispute are social justice, progress, the role of innovation and technology, community, rights, duties and personal freedom.

These disputes are not unfortunate; they are integral to a pluralistic society. Decisions cannot be made effectively without recognizing and addressing them. Knowledge of the natural world (i.e. science) will narrow the range of reasonable options. But, science does not offer insights or methodologies to hasten resolution between conflicting value systems. In fact, the attempt to resolve these debates with appeals to science alone is often a diversion to avoid addressing emotionally charged debates on values [18,19]. Given the large degree of scientific uncertainty in the interpretation of environmental biomonitoring data, and confronted with strongly conflicting beliefs and values, how can we reach effective decisions?

### Argumentation

Argumentation (sometimes called "Rhetoric") is a process to reach decisions through reasoned persuasion rather than fiat or whim [20-22]. Unlike the scientific method, which examines the universal and constant laws of nature, argumentation applies reason to the particular and contingent realm of human affairs. It is a process to discover workable resolutions, and in its broadest definition, includes both "normative" and "non-normative" arguments [23]:

• "Normative" arguments employ "high-minded" principles that the participants have agreed to apply. The parties agree to be open-minded and willing to change their initial position. Positions (arguments) are supported by facts and reason. These processes are ambitious and time consuming because they ask the participants to set aside competing self-interests and deeply held beliefs.

• "Non-normative" processes involve negotiation or bargaining to reach an acceptable resolution. There is no attempt to agree upon values or principles. These are adversarial processes dominated by self-interest. Many of life's daily tasks, especially in commercial transactions, are accomplished by non-normative rules. These processes appeal to authority, socially sanctioned beliefs, the status quo and emotion. When necessary, they employ guile, deception, and the advantage of proprietary information. These behaviors are legitimate, efficient and sanctioned in all societies – as long as the parties understand the rules in play.

### Decisions that impact human health

Many societies believe that decision-making in the area of human health should be guided by normative frameworks because:

• Health is a fundamental "good" that should not be subject to negotiation – especially involving the weak, vulnerable and uniformed

• Unlike most harm or damages in life, human health cannot always be reversed, nor adequately compensated

The willingness to accept a normative framework (in this case, bioethical principles) for making decisions in the arena of human health is, in itself, unsupported by any natural or scientific knowledge. It's a value based decision, and a *sine quo non *for participation in a process of bioethical argumentation.

### Applying bioethical principles is a normative argumentation process

Bioethical principles have been employed with relative success in many contentious and value-laden health controversies [24,25]. The important role that this approach has played in developing public policy around the use of genetic information was mentioned previously. Other areas of productive application are procreation and the right to reproduction, protection of human subjects in research, life support, euthanasia, abortion, stem cell research, access to health care, and discrimination based upon disability. None of these disputes is ever wholly resolved, but bioethical principles, in concert with other tools such as casuistry (case precedents) have provided a framework for productive dialogue [26].

### What are the "conventional bioethical principles"?

"Conventional bioethical principles," have gained wide use for evaluating policies, programs or activities that may entail risk to human health. This is because they "work" in the real world. The four major ethical principles in bioethics are viewed as duties that many contemporary philosophers believe to be *prima facie*. *Prima facie *duties take precedence over any other considerations except another duty. The "big four" are:

• "Autonomy," also known as the "respect for humans" principle, acknowledges the belief that an individual understands his or her own best interests better than anyone else

• "Beneficence" means to "do good" for people; all stake holders are to be considered

• "Nonmaleficence," sometimes seen as a corollary to beneficence, means to "do no harm" to people

• "Justice" captures the belief that there should be a fair distribution of the benefits and costs (including risks to health) of an activity or program

Beauchamp and Walters list four additional bioethical principles which they refer to as "secondary principles" [24]. The one that most often comes into ethics discussions is veracity. A normative process cannot proceed in the face of disingenuous interpretations of scientific knowledge and other established truths.

• "Utility" describes the idea that actions should achieve the most good for the greatest number of people.

• "Fidelity" means that decisions regarding controversies should demonstrate consistency with other similar cases

• "Veracity" holds that decisions or policies should neither ignore established truths nor try to state beliefs as such

• "Confidentiality" is the idea that an individual's right to privacy should be protected

### How do principles work?

Making decisions guided by these principles is an iterative dialogue between parties, the more culturally and philosophically diverse the better. The principles are not viewed as rigid rules or prohibitions, but provide a vocabulary and useful "warrants" during argumentation. Decisions require practical compromises that are highly dependent upon context, precedents, and a well-honed sense of the "possible" [27].

Different, equally talented groups can come to different conclusions when faced with identical problems whether in an Institutional Review Board (IRB) or a public policy discussion. In fact; the same group can deliver different answers on different days. This isn't surprising if one recognizes that difficult decisions do not typically address "right versus wrong". Difficult decisions invariably result when the choices involve "right versus right" [28]. Some important points to make about bioethical argumentation are:

• Bioethical principles offer guidance, but no absolutes.

• Principles often conflict; no principle is preeminent.

• Bioethical principles are not employed in argumentation at the exclusion of other equally powerful "tools," notably casuistry.

• No activity or program is inherently ethical or unethical; they are judged by balancing benefits and risks to all stakeholders while guided by principles.

• Any form of deception is viewed as inherently unethical in normative deliberations.

• The specific use of biomonitoring data determines the principles that are most germane; this discussion arbitrarily groups the uses into four areas – human research, public health, product stewardship and medical practice.

## Discussion

### Human research

Research designed to validate or employ biomarkers for exposure does not create any unique issues. The application of bioethical principles to human research is highly developed and codified in documents such as the "Declaration of Helsinki" [29], the "International Ethical Guidelines for Biomedical Research Involving Human Subjects" [30] and in regulations such as the U. S. "Common Rule" [31]. Protections such as informed consent, written protocols and publication of all findings are widely practiced under the purview of IRBs in the U.S. and Regional Ethics Committees and Institutional Ethics Committees in other parts of the world [32,33].

The principles have been used to address other considerations in the conduct of research besides direct harm to participants. Notably, there is increasing interest on the part of IRB members in the scientific validity of proposed research. The rationale is simple: if the design of proposed research does not offer the prospect of yielding objective and scientifically valid conclusions, there is no justification for either the expenditure of resource or subjecting participants to even *de minimis *risk. Research protocols that are fundamentally flawed cannot be justified in the light of the principles of beneficence and veracity. A recent review on proposed research using pesticides makes the point well in terms of accepting research results for rule-making.

"Any study that is not scientifically valid – for example, does not include a sufficient number of subjects to provide statistically valid answers to the questions under investigation -must not be considered in standard setting" [34]

Bioethical principles go further and support the argument that these decisions should be made before the research is initiated. Poorly designed studies involving human subjects should not be undertaken.

Another conflict that has arisen with human biomarkers is the desire of research participants to learn their individual results. There is no easy answer, and two principles, nonmaleficence and autonomy, seem to give different answers. A careful balancing of risks and benefits by the investigators may argue against sharing individual data in the early stages of investigating a biomarker. This is an ethically supportable position as long as the research participants are informed of the decision to not disclose before participation. A precedent for this position has been established in genetic research because of the psychological and financial harm that can ensue from misinformation on health risk [35]. Another view of this issue, described as the "community-based participatory research" approach emphasizes the "right to know" (autonomy), and argues that individual data should routinely be shared with research participants [9]. This position is not unreasonable, but the facts, context and stakeholders in specific cases could support different decisions.

The U.S. Center for Disease Control's National Exposure Report is a good example of how biomonitoring information can be generated and communicated with high scientific and ethical standards [10]. Individual test results are not given unless the concentration is markedly above population means. Notification in these rare cases is ethically justified because an individual may be able to identify the source of unusual exposure and correct it. The principle of beneficence argues for disclosure. At the same time, the CDC is careful to emphasize that mere presence of the chemical, even at multiple standard deviations above the group mean does not imply health risk (the principles of nonmaleficence and veracity are thereby satisfied).

One of the important ethical questions in the area of human research is why many organizations do not adopt frameworks to protect human subjects and to ensure that the research has scientific value. Though this is not legally required for privately funded research, the failure of organizations to voluntarily adopt the Common Rule or a similar standard of behavior raises questions about the integrity of their research programs. Multiple bioethical principles support arguments against the conduct of human research without appropriate protections (e.g. autonomy, beneficence, utility and veracity) [36].

The area of human research has served as the beachhead for the application of bioethics to real world societal controversies. Difficult decisions in areas besides research can also benefit from disciplined argumentation that employs these processes and principles.

### Public health including surveillance

Surveillance activities are designed to detect environmental conditions that increase the risk of adverse health outcomes with the purpose of controlling or eliminating those conditions [37]. Conceptually, surveillance does not address individual risk, though the same data can be used to do so (medical screening).

Using biomonitoring data as a surveillance tool is already contributing to improved public health protections. Examples are:

• monitoring time trends of chemical concentrations in the population to better target testing

• validating the effectiveness of regulation

• targeted assessment of populations suspected to be in environmental "hotspots"

• investigating "clusters" of disease that might be related to an environmental exposure

• detecting emerging exposures that were unsuspected

From a bioethical perspective, any reasoned analysis seems to argue for a very broad application of biomonitoring technology for the purposes above. Surveillance, which by definition, is not engaged in research, should use only validated biomarkers. Also, because surveillance looks at group analyses, the thorny issues that accompany individual results can largely be avoided.

There is a long history of using exposure biomarkers in the workplace, though its application has been much more extensive in Europe than in North America. The American Council of Governmental and Industrial Hygienists has developed Biological Exposure Indices (BEIs) for approximately 50 chemicals [38]. These guidelines are designed to be used by industrial hygienists in making decisions regarding safe levels of exposure to chemical substances in the workplace. German Biological Tolerance Values (BAT values) are similar, but derived for medical screening and diagnosis purposes [39]. There are significant regulatory and work council constraints in the occupational setting that do not apply to biomonitoring for environmental exposure. These formal rules ensure the validity of the analyses, and protect against breach of confidentiality and discrimination in employment or other benefits. Absent these protections and those of the Common Rule, participants in environmental biomonitoring depend upon the ethical sophistication and good intentions of the administrators of these programs.

A significant challenge for these state-of-the-art analyses is that they must be done competently. This is not easy [40]. The measurements are being done on very low concentrations of analyte, there is little standardization of the methods and there is no independent process for assuring the quality of the analyses. Large, experienced labs, such as the one at the National Center for Environmental Health have the skills, resources and quality control systems to optimize the validity of the results. But the measurement of these biomarkers is done largely by small unaccredited labs. Issues of sample collection, storage and transport are also major challenges. It is profoundly unethical to communicate data used for human health decisions unless all efforts have been taken to assure its integrity. Even then the result should be communicated with accurate descriptions of the test validity.

Another bioethical consideration arises when specific communities are selected for testing. The rationale may be very sound, but the risk of stigmatizing an ethnic, geographical or professional community needs to be discussed. This will become more salient if and when the low concentrations of environmental chemicals being detected to date are linked to increased health risk. Even then, nonmaleficence does not necessarily take precedence over potential benefits, but this consideration needs to be balanced against the anticipated benefits.

### Regulatory risk assessment is an important element of public health practice

Biomonitoring information is currently not employed in these processes because there is not enough factual knowledge about the linkage between a specific biomarker of absorption and human disease [41,42]. Exposure biomarkers will augment, and in some cases, supplant exposure estimates for the purpose of risk assessments, but the validation work is yet to be done.

In the near term, biomarkers of exposure can be linked to established human reference values for environmental exposure with physiologically based pharmacokinetic (PBPK) information [43]. This process enabled the development of workplace BEIs discussed above. The necessary PBPK information – which describes the biology of how a chemical is absorbed, distributed, metabolized and excreted – exists for no more than 100 chemicals. That information makes it possible to model the relationship between a biomonitoring concentration and existing health-based criteria such as reference doses (RfDs) or tolerable daily intakes (TDIs). Calculating the concentration of a "biomonitoring equivalent" (BE) yields a screening tool that enables a qualified statement on population risk. The main ethical challenge will be recognize the technical uncertainties in these derivations, and to not overstate the meaning of a measurement in terms of either safety or increased risk for an individual.

### Product stewardship

Product stewardship is a public health activity that requires its own analysis because it is the responsibility of the private sector. This gives the organizations involved much greater latitude in terms of strategy and resources as they do not have to justify their actions to politicians and the public. At the same time, the responsibility creates significant normative ethical expectations that are not clearly defined.

Product stewardship decisions rely upon assessments of risk. The area of greatest uncertainty in these is exposure estimation – a methodology generously described as an educated guess. The availability of human biomonitoring technologies creates an opportunity for product stewards to understand and utilize its capabilities. The techniques should be employed if they are likely to yield a more accurate assessment of risk to customers and the community. It is likely that this will not be necessary in most cases. But, this decision can be made only after a systematic assessment of the potential contribution of exposure biomonitoring for each product or emission. All of the bioethical principles listed above are applicable to these decisions.

### Medical practice

The use of population-based biomonitoring information in risk assessment and other public health applications raises fewer ethical objections than the use of the same data when it is personally identified. The latter routinely leads into the realms of medical screening and medical diagnosis where well-intentioned efforts can cause great harm.

Medical diagnosis and treatment occurs in a special relationship – that of physician and patient. A symptomatic individual [a patient] initiates that relationship when they ask for help. The physician is ethically and legally committed to a fiduciary role in which he or she puts the best interests of the patient above other considerations. At a minimum, the practitioner is expected to be adequately trained, licensed and knowledgeable about the tests and therapies that he or she employs. This includes understanding the scientific rationale of any biomarkers in order to be able to interpret and act on the information. Given the current paucity of knowledge about the biological significance of environmental chemicals at the tissue concentrations that have been documented, it is highly unlikely that a responsible physician will link these doses to a disease, nor recommend any treatment or preventive actions. To do so risks harming the patient in multiple ways – physically, psychologically and financially – without any evidence of likely benefit. The legal, professional, financial and reputational sanctions for this type of breach of a physician's duty are severe. While we can readily apply the principles of autonomy, veracity, beneficence and nonmaleficence to an analysis of this decision, it may well be the more pedestrian principle of enlightened self-interest that proves to be most persuasive with medical practitioners.

The use of biomarkers of environmental exposure to accomplish medical screening (very different in concept than either surveillance or diagnosis) certainly strays farther from the "moral sphere" than anything discussed to this point. Screening is an activity in which an individual without any symptoms is tested for early signs of a disease. While some screening tests [and screening programs] are scientifically and ethically sound, many of these activities meet neither test. There are many well documented reasons for this, but, in short, there is a fundamental inability of most tests to predict future presence or absence of disease [44,45].

In the case of current biomarkers of environmental exposure, it is difficult to imagine that the "screeners," believe that they are benefiting individuals by sharing personal test data. The principle of autonomy supports the "right to know," but the principles of beneficence, nonmaleficence and veracity seem to support nondisclosure. One reason is that it is highly unlikely that an individual gains any benefit in knowing his personal test results as opposed to the readily available group mean given to the community (certainly, valuable knowledge). This statement assumes that, as is the case with the CDC's programs, any notably high numbers are reported routinely to the participant.

Is there any "actionable" information in personal data from routine environmental biomonitoring? The answer to this question provides the kind of factual context ("specification" in the language of Tom Beauchamp) that allows practical guidance to be drawn out of general moral principles [46]. And, he adds, don't forget the value and authority of "paradigmatic cases," i.e. casuistry, which works hand in glove with bioethical principles. In the case of harm done to individuals by medical screening there is a very rich literature [47,48].

## Conclusion

It is often said that the ability to measure chemicals in humans is far outpacing the ability to interpret these data for public and individual health purposes. Future research will yield evermore knowledge of the biology that links exposure to effect, and will reduce the uncertainties. Even then, we will need a way to resolve substantive disagreements in the meaning of this data that are matters of belief rather than knowledge. Both now and in the future, a normative argumentation framework, using bioethical principles in conjunction with other practical tools such as casuistry, offers a reasonable approach to improving decisions that involve human health impacts. Not only does the process surface important disagreements in values, but when done well, illuminates unsupportable interpretations of science.

"Engaging in the steps of an ethics analysis makes us meticulous in our reasoning, requiring us to advocate interventions on the basis of facts and not merely belief. Further, an ethics analysis holds us to high standards, not only for scientific method, but also for how respectfully we communicate with and involve constituent communities." [49]

## Competing interests

The author declares that they have no competing interests.

## References

[B1] National Research Council (2006). Human Biomonitoring for Environmental Chemicals.

[B2] EBSIO Development of a coherent approach to human biomonitoring in Europe Coordination Action Priority 8.1 PUBLISHABLE FINAL ACTIVITY REPORT. http://www.eu-humanbiomonitoring.org/doc/rep_far.pdf.

[B3] European Center for Ecotoxicology and Toxicology of Chemicals (2005). Guidance for the Interpretation of Biomonitoring Data.

[B4] U.S. President's Commission for the Study of Ethical Problems in Medicine and Biomedical and Behavioral Research (1983). Screening and Counseling for Genetic Conditions.

[B5] Rothstein MA, (Ed) (1991). Legal and Ethical Issues Raised by the Human Genome Project: Proceedings of the Conference in Houston, Texas, 7–9 March 1991.

[B6] Schulte PA, Schulte, Perera (1993). A Conceptual and Historical Framework for Molecular Epidemiology. Molecular Epidemiology: Principles and Practices.

[B7] Rosenberg J, Rempel D (1990). Biological Monitoring. Occupational Medicine State of the Art Reviews.

[B8] Barrett JC (1997). 12th Meeting of the Scientific Group on Methodologies for the Safety Evaluation of Chemicals: Susceptibility to Environmental Hazards. Environmental Health Perspectives.

[B9] Brody JG (2007). Improving Disclosure and Consent: "Is It Safe?": New Ethics for Reporting Personal Exposures to Environmental Chemicals. American Journal of Public Health.

[B10] Centers for Disease Control and Prevention [CDC] (2005). National Report on Human Exposure to Environmental Chemicals.

[B11] Sexton K (2004). Human biomonitoring of environmental chemicals; measuring chemicals in human tissues is the "gold standard" for assessing exposure to pollution. American Scientist.

[B12] Paustenbach D, Galbraith D (2006). Biomonitoring and Biomarkers: Exposure Assessment Will Never Be the Same. Environmental Health Perspectives.

[B13] (2007). Our Stolen Future. http://www.ourstolenfuture.com/NewScience/oncompounds/bodyburden/2003-0131-CDC-bodyburden.htm.

[B14] (2007). WWF-UK. http://www.wwf.org.uk/chemicals/survey/introduction.asp.

[B15] Environmental Working Group, Body Burdens (2005). The Pollution in Newborns Washington DC.

[B16] Committee on Toxicity and Assessment of Environmental Agents, National Research Council (2007). Toxicity Testing in the Twenty-first Century: A Vision and Strategy Washington DC.

[B17] Goldman S (2006). Science Wars: What Scientists Know and How They Know It.

[B18] Wagner WE (1995). The Science Charade in Toxic Risk Regulation. Columbia Law Review.

[B19] Sarewitz D (2004). How Science Makes Environmental Controversies Worse. Environment, Science and Policy.

[B20] Zarefsky D (2001). Argumentation: The Study of Effective Reasoning.

[B21] Toulmin S (1958). The Uses of Argument.

[B22] Perelman C (1982). The Realm of Rhetoric.

[B23] Provis C (2004). Negotiation, Persuasion and Argument. Argumentation.

[B24] Beauchamp T, Walters L, (Eds) (1994). Contemporary Issues in Bioethics.

[B25] Veatch RM (1997). Medical Ethics.

[B26] Brody BA (1998). The Ethics of Biomedical Research.

[B27] Toulmin S (1981). The Tyranny of Principles. Hastings Center Report.

[B28] Badaracco JL (1997). Defining Moments: When Managers Must Choose Between Right and Right.

[B29] World Medical Association (1997). Declaration of Helsinki. JAMA.

[B30] Council of International Organizations of Medical Sciences (1993). International Ethical Guidelines for Biomedical Research Involving Human Subjects Geneva.

[B31] Department of Health and Human Services [DHHS] (1991). Title 45 Code of Federal Regulations Part 46 Protection of Human Subjects.

[B32] Department of Health (1991). Local Research Ethics Committees (HSG(91)5) London.

[B33] Holm SO (2007). The Danish Research Ethics Committee System, Overview and Critical Assessment Research Involving Human Participants V2.

[B34] Oleskey C (2004). Pesticide Testing in Humans: Ethics and Public Policy. Environmental Health Perspectives.

[B35] Soskolne CL (1997). Ethical, Social and Legal Issues Surrounding Studies of Susceptible Populations and Individuals. Environmental Health Perspectives.

[B36] MacDonald C (2004). Higher standards for privately funded health research. Canadian Bioethics Society Newsletter.

[B37] Last JM (1983). A Dictionary of Epidemiology.

[B38] American Conference of Governmental and Industrial Hygienists (2007). Threshold Limit Values for Chemical Substances and Physical Agents and Biological Exposure Indices Cincinnati, Ohio.

[B39] Deutsche Forschungsgemeinschaft (DFG) (Eds) (2007). List of MAK and BAT Values 2007: Maximum Concentrations and Biological Tolerance Values at the Workplace Bonn.

[B40] Bates MN (2005). Workgroup Report: Biomonitoring Study Design, Interpretation, and Communication – Lessons Learned and Path Forward. Environmental Health Perspectives.

[B41] Doerrer NG, Holsapple MP (2004). Integration of biomonitoring exposure data into the risk assessment process. Risk Policy Report.

[B42] Angerer J (2006). Strategic Biomonitoring Initiatives: Moving the Science Forward. Toxicological Sciences.

[B43] Hays SM, Becker RA, Leung HW, Aylward LL, Pyatt DW (2007). Biomonitoring equivalents: a screening approach for interpreting biomonitoring results from a public health risk perspective. Regul Toxicol Pharmacol.

[B44] Wilson JMG, Jungner G (1968). Principles and practice of screening for disease.

[B45] Berg AO, Allan JD (2001). Introducing the Third U.S. Preventive Services Task Force. Am J Prev Med.

[B46] Shickle D, Chadwick R (1994). The ethics of screening: Is "screeningitis" an incurable disease?. Journal of Medical Ethics.

[B47] Sackett DL, Holland WW (1975). Controversy in the detection of disease. Lancet.

[B48] Beauchamp TL (2003). Methods and Principles in Biomedical Ethics. J Med Ethics.

[B49] Kass NE (2001). An Ethics Framework for Public Health. AJPH.

